# Supplementary material for: Content-rich biological network constructed by mining PubMed abstracts
Source: BMC Bioinformatics. 2004 Oct 8;5:147. doi: 10.1186/1471-2105-5-147 (PMC528731; doi:10.1186/1471-2105-5-147)
Supplement: Additional File 2 — The original results of the above study (non-essential files are deleted to keep the file size under the limit set by BMC bioinformatics). [file 1471-2105-5-147-S2.bz2 › chilibotAdditionalFile2/dip40/704ID10734142E18041/html/MUTL_MUTS.html]

 


 **MUTL** and **MUTS** 
  
Found 186 abstracts in PubMed, retrieved 40.  
 

 What does Google say? 
 PDF only 
| .edu only 

---

**Interactive relationship** (e.g. stimulation, inhibition, etc)

**Neutral relationship**- Such a higher order  **MutS**  complex productively interacts with  **MutL**  in ATP hydrolyzing conditions and generates a specific ternary complex, which might communicate with MutH.  Ref: 11888282 Biochemistry, 2002
- Human mismatch repair protein  **MutL**  homologue 1 MLH1 interacts with Escherichia coli  **MutL**  and  **MutS**  in vivo and in vitro a simple genetic system to assay MLH1 function.  Ref: 12513688 Biochem J, 2003
- The pathway in eukaryotes consists of the DNA binding components, which are the homologs of the bacterial  **MutS**  protein MSH 2 6, and the  **MutL**  homologs, which bind to the  **MutS**  homologs and are essential for the repair process.  Ref: 12091911 Nat Genet, 2002
- Both pathways require the formation of a  **MutS**  homolog complex which interacts with a second heterodimer, composed of two  **MutL**  homologs.  Ref: 12836196 Med Sci Paris , 2003
- **MutS**  initiates repair by binding to the mismatch, and activates together with  **MutL**  the MutH endonuclease, which incises at hemimethylated dam sites and thereby mediates strand discrimination.  Ref: 11920679 J Cell Physiol, 2002
- **MutL**  related proteins were also required for mutation avoidance in stationary phase cells, but to a lesser extent than  **MutS**  homologues.  Ref: 12491007 Curr Genet, 2002
- In other bacteria, the requirement for sequence identity during recombination is partly due to the actions of the mismatch repair system, a key component of which,  **MutS** , recognizes mismatched bases in heteroduplex DNA and, along with  **MutL** , blocks strand exchange.  Ref: 11698371 J Bacteriol, 2001

**Non-interactive relationship** (e.g. studied together, co-existance, homology, etc.)

- As compared to native  **MutS** , the ability of MutS800 to promote  **MutL**  dependent activation of MutH is substantially reduced.  Ref: 12829697 J Biol Chem< MedlineTA>J Biol Chem, 2001
- In eukaryotes, homologs of the Escherichia coli  **MutS**  and  **MutL**  proteins are crucial for both meiotic recombination and post replicative DNA mismatch repair.  Ref: 12836196 Med Sci Paris , 2003
- The average percentages of positively stained cells for human  **MutL**  homolog 1 hMLH1, human  **MutS**  homolog 2 hMSH2, and human  **MutS**  homolog 6 hMSH6 in RGP CMM 75.6 3.4%, 67.20 7.71%, and 76.6 2.1%, respectively were reduced compared with benign nevi.  Ref: 12112321 Mol Carcinog, 2002
- These effects parallel the  **MutL**  and  **MutS**  binding and ATP binding hydrolysis activities of the mutated proteins.  Ref: 12513688 Biochem J, 2003
- The products of  **mutS**  and  **mutL** , which are required for adenine methylation directed mismatch repair MMR, enhance VSP repair.  Ref: 11591694 J Bacteriol, 2001
- However, several  **MutS**  like and  **MutL**  like proteins also participate in meiotic recombination.  Ref: 12095912 Hum Mol Genet, 2002
- We also describe the alterations in the  **mutS** ,  **mutL**  and uvrD genes responsible for the mutator phenotype of hypermutable P. aeruginosa strains isolated from CF patients.  Ref: 11952911 Mol Microbiol, 2002
- Following a common mismatch recognition step effected by  **MutS**  homologous Msh proteins, homologs of the bacterial  **MutL**  ATPase predominantly the Mlh1p Pms1p heterodimer in yeast couple mismatch recognition to the appropriate downstream processing steps.  Ref: 12454061 Genetics, 2002
- Strand specificity and repair efficiency are largely independent of the GATC methylation state of the DNA and presence of the products of mismatch repair genes mutH,  **mutL** , and  **mutS** .  Ref: 12692132 J Biol Chem< MedlineTA>J Biol Chem, 2002
- MutH,  **MutL**  and  **MutS**  are essential components of the mismatch repair system in Escherichia coli.  Ref: 11934505 FEMS Microbiol Lett, 2002
- We have analysed the levels of mRNA transcripts of the  **mutS**  and  **mutL**  homologous genes of the yeast Saccharomyces cerevisiae during the course of meiosis, by quantitative RT PCR.  Ref: 11523800 Mol Genet Genomics, 2001
- Protein components responsible for these reactions are highly conserved through evolution, and homologs of bacterial  **MutS**  and  **MutL** , which are key players in the initiation steps of both the strand specific mismatch correction and MMR dependent apoptotic signaling, have been identified in human cells.  Ref: 12700127 Front Biosci, 2003
- Transcription of  **mutS**  and  **mutL**  homologous genes during meiosis in Saccharomyces cerevisiae and identification of a regulatory cis element for meiotic induction of MSH2.  Ref: 11523800 Mol Genet Genomics, 2001
- Exonuclease 1 Exo1 is a 5 3 exonuclease that interacts with  **MutS**  and  **MutL**  homologs and has been implicated in the excision step of DNA mismatch repair.  Ref: 12629043 Genes Dev, 2003
- The results shown here indicate that the putative P. aeruginosa  **mutS** ,  **mutL**  and uvrD genes are mutator genes.  Ref: 11952911 Mol Microbiol, 2002
- To address these questions, mice with inactivating mutations in all the known  **mutS**  and  **mutL**  homologs have been generated.  Ref: 12114115 Trends Mol Med, 2002
- iv defects in  **mutL**  or  **mutS**  appeared to have a stronger effect on growth dependent mutations than on adaptive mutations.  Ref: 11733076 Mutat Res, 2001
- RESULTS All cell lines examined showed decreased levels of  **MutS**  and  **MutL**  DNA MMR proteins in a dose dependent manner after coculture with H. pylori strains.  Ref: 12145807 Gastroenterology, 2002
- **MutS**  hMSH2 and hMSH6 and  **MutL**  hMLH1, hPMS2, and hPMS1 DNA MMR protein and RNA levels were determined.  Ref: 12145807 Gastroenterology, 2002
- We analysed the expression of two mismatch repair proteins, human  **mutL**  homologue 1 hMLH1 and human  **mutS**  homologue 2 hMSH2, and evaluated the frequency of microsatellite instability with 10 mononucleotide and two dinucleotide repeat sequences, in 39 paired tumour normal DNA samples obtained from 17 testicular and two mediastinal germ cell tumours.  Ref: 11719586 Mol Hum Reprod, 2001
- Multiple  **MutS**  and  **MutL**  homologues exist in eukaryotes, which play different roles in the mismatch repair MMR pathway or in recombination.  Ref: 11920679 J Cell Physiol, 2002
- **MutS**  and  **MutL** , part of the post replication mismatch repair system, stimulate VSP repair.  Ref: 11585365 Mutat Res, 2001
- To investigate the effects on PV rates of mutations in genes for mismatch repair MMR, insertion deletion mutations of  **mutS** ,  **mutL** , mutH, dam, polI, uvrD, mfd and recA were constructed in Hi strain Rd.  Ref: 11889052 EMBO J, 2002
- At a low dose, dP is much more mutagenic in mismatch repair defective mutH,  **mutL** , and  **mutS**  strains than in a wild type strain.  Ref: 12196386 Genetics, 2002
- BACKGROUND The aim of this study was to search for mutations in the human  **mutS**  homolog 2 hMSH2 and human  **mutL**  homolog 1 hMLH1 genes in 25 unrelated Brazilian kindreds with suspected hereditary nonpolyposis colorectal cancer HNPCC.  Ref: 12095971 Ann Surg Oncol, 2002
- The mismatch repair MMR system plays a central role in maintaining genetic stability and requires evolutionarily conserved protein factors, including  **MutS**  and  **MutL**  homologs.  Ref: 12095912 Hum Mol Genet, 2002
- We also show that interaction between Vsr and  **MutL**  inhibits the ability of  **MutL**  to dimerize, to interact with  **MutS**  and MutH and to mediate a previously unknown interaction between  **MutS**  and MutH.  Ref: 11585365 Mutat Res, 2001
- The human mismatch repair hMMR system consists of members of three super families, namely MutH,  **MutL**  and  **MutS** .  Ref: 12578652 Zhongguo Shi Yan Xue Ye Xue Za Zhi< MedlineTA>Zhongguo Shi Yan Xue Ye Xue Za Zhi, 2001
- Specific ternary complexes of  **MutS**   **MutL**  heteroduplexes were formed only during ATP hydrolysis.  Ref: 11888282 Biochemistry, 2002
- In vitro and in vivo studies of  **MutS** ,  **MutL**  and MutH mutants correlation of mismatch repair and DNA recombination.  Ref: 12606120 DNA Repair Amst , 2003
- Until now, a number of works have focused on the function of  **MutS**  and  **MutL**  homologs during mismatch DNA repair.  Ref: 11553915 Mol Reprod Dev, 2001
- The introduction of mismatches by growth in 2 aminopurine caused a replication dependent increase in the number of cells with  **MutS**  and  **MutL**  foci.  Ref: 11779496 Mol Cell, 2001
- The mutator phenotype was due to the ability of hMLH1 protein to interact with bacterial  **MutL**  and  **MutS**  proteins, thereby interfering with the formation of complexes between MMR proteins and mismatched DNA.  Ref: 12513688 Biochem J, 2003
- An unsolved problem in E. coli mismatch repair is how the  **MutS**   **MutL**  complex communicates positional information of a mismatch to MutH.  Ref: 11888282 Biochemistry, 2002
- In order to understand the role of the yeast mismatch repair MMR system in the mutagenesis of stationary phase cells, we studied the effects of deletions in genes encoding  **MutS**  and  **MutL**  related proteins on the reversion frequency of the lys2 Delta Bgl frameshift mutation.  Ref: 12491007 Curr Genet, 2002
- Seven out of the 11 mutator strains were found to be defective in the MMR system four  **mutS** , two  **mutL**  and one uvrD.  Ref: 11952911 Mol Microbiol, 2002
- Finally, every mutation that abolishes mismatch repair is deficient in blocking homeologous recombination, suggesting that mismatch repair and prevention of homeologous recombination use the same  **MutS**   **MutL**  complexes for signaling in E. coli.  Ref: 12606120 DNA Repair Amst , 2003
- Whereas  **mutS**  and  **mutL**  genes are found in most organisms, the mutH gene is limited to some proteobacteria.  Ref: 11934505 FEMS Microbiol Lett, 2002
- Since the discovery of a link between the malfunction of post replicative MMR and human cancers, a number of works have focused on the function of  **MutS**  and  **MutL**  homologs in the correction of replication errors.  Ref: 12095912 Hum Mol Genet, 2002
- The  **MutS**  homologous MSH and  **MutL**  homologous MLH proteins are highly conserved among all prokaryotes and eukaryotes.  Ref: 12582547 Theor Appl Genet< MedlineTA>Theor Appl Genet, 2002
- We have used the recently determined crystal structures of Escherichia coli E. coli  **MutS** ,  **MutL**  and MutH to guide construction of 47 amino acid substitutions in these proteins and analyzed their behavior in mismatch repair and recombination in vitro and in vivo.  Ref: 12606120 DNA Repair Amst , 2003
- In four cases three  **mutS**  and one  **mutL** , the genes contained frameshift mutations.  Ref: 11952911 Mol Microbiol, 2002
- In eukaryotic cells, DNA mismatch repair is initiated by a conserved family of  **MutS**  Msh and  **MutL**  Mlh homolog proteins.  Ref: 12529393 Mol Cell Biol, 2003
- **MutS**  and  **MutL**  homologs have been implicated in multiple genetic stabilization pathways.  Ref: 11717437 Proc Natl Acad Sci U S A, 2001
- **MutL**  GFP formed foci in a subset of cells, and its localization was  **MutS**  dependent.  Ref: 11779496 Mol Cell, 2001
- VSP repair and MMR share two proteins,  **MutS**  and  **MutL** , and although the two repair pathways are not equally dependent on these proteins, their dual use creates a competition within the cells between the repair processes.  Ref: 12067333 Mol Microbiol, 2002
- These results implicate  **MutS**  alpha and  **MutL**  alpha in the suppression of gene amplification and suggest that defects in this genetic stabilization function may contribute to the cancer predisposition associated with mismatch repair deficiency.  Ref: 11717437 Proc Natl Acad Sci U S A, 2001
- The  **mutS**  and  **mutL**  genes, which are contiguous in the L. monocytogenes chromosome, were identified after in silico analysis.  Ref: 11994166 Mol Microbiol, 2002
- The mismatch repair system  **mutS** ,  **mutL**  and uvrD genes in Pseudomonas aeruginosa molecular characterization of naturally occurring mutants.  Ref: 11952911 Mol Microbiol, 2002
- The DNA repair genes  **mutL**  and  **mutS**  were separated by the prophage remnant SF370.4.  Ref: 12441069 Virology, 2002
- the cause of MMR deficiency in these cell lines is due to a defective  **MutS**  alpha or  **MutL**  alpha.  Ref: 12173046 Oncogene, 2002
- Evidence to support a role for the mismatch repair genes human  **mutL**  homolog 1 hMLH1 and human  **mutS**  homolog 2 hMSH2 in the etiology of colorectal cancer has come from linkage analysis, segregation studies, and molecular biologic analysis.  Ref: 12419761 Am J Epidemiol, 2002
- The mammalian DNA mismatch repair genes belong to a family of genes that comprise several homologs of the Escherichia coli  **mutS**  and  **mutL**  genes.  Ref: 12114115 Trends Mol Med, 2002
